# Reliance on model-based and model-free control in obesity

**DOI:** 10.1038/s41598-020-79929-0

**Published:** 2020-12-31

**Authors:** Lieneke K. Janssen, Florian P. Mahner, Florian Schlagenhauf, Lorenz Deserno, Annette Horstmann

**Affiliations:** 1 Integrated Research and Treatment Center Adiposity Diseases, Leipzig University Medical Center, Leipzig, Germany; 2 Department of Neurology, Max Planck Institute for Human Cognitive and Brain Sciences, Leipzig, Germany; 3 Department of Psychiatry and Psychotherapy, Charité-Universitätsmedizin Berlin, Campus Charité Mitte, Berlin, Germany; 4 Max Planck UCL Centre for Computational Psychiatry and Ageing Research, University College London, London, UK; 5 The Wellcome Centre for Human Neuroimaging, University College London, London, UK; 6 Department of Child and Adolescent Psychiatry, Psychotherapy and Psychosomatics, University of Würzburg, Würzburg, Germany; 7 Department of Psychology and Logopedics, Faculty of Medicine, University of Helsinki, Helsinki, Finland

**Keywords:** Decision, Learning algorithms, Reward, Human behaviour

## Abstract

Consuming more energy than is expended may reflect a failure of control over eating behaviour in obesity. Behavioural control arises from a balance between two dissociable strategies of reinforcement learning: model-free and model-based. We hypothesized that weight status relates to an imbalance in reliance on model-based and model-free control, and that it may do so in a linear or quadratic manner. To test this, 90 healthy participants in a wide BMI range [normal-weight (n = 31), overweight (n = 29), obese (n = 30)] performed a sequential decision-making task. The primary analysis indicated that obese participants relied less on model-based control than overweight and normal-weight participants, with no difference between overweight and normal-weight participants. In line, secondary continuous analyses revealed a negative linear, but not quadratic, relationship between BMI and model-based control. Computational modelling of choice behaviour suggested that a mixture of both strategies was shifted towards less model-based control in obese participants. Our findings suggest that obesity may indeed be related to an imbalance in behavioural control as expressed in a phenotype of less model-based control potentially resulting from enhanced reliance on model-free computations.

## Introduction

Obesity is the result of systematically consuming more energy than is expended. This can be seen as a failure of control over eating behaviour^1–3^ and could result from altered processing of reward^4^. As a consequence, appetitive and often high-caloric foods are over-consumed despite negative consequences, such as the uncomfortable feeling of being full, feelings of regret, or long-term health risks. Such failures of behavioural control in obesity may arise from alterations in reinforcement learning^5^. Indeed, obesity-related impairments in reward- and punishment-based cue-conditioning have been observed in the context of both food and monetary outcomes^6^, as well as impairments in appetitive conditioning in the context of chocolate rewards^7^ (but see^8^). Furthermore, obese participants exhibited impairments in learning from negative outcomes when money or points served as an incentive^6,9,10^. These studies have focused on forms of learning that mostly resemble retrospective model-free ‘trial-and-error’ reinforcement learning. However, behavioural control arises from a balance between model-based and model-free control^11,12^. Model-based control relies on an internal model of the environment to enable forward planning. As a result, this system is flexible (but cognitively costly), allowing us to be goal-directed even when the environment changes, e.g. abrupt change in the current outcome value, changes. In contrast, the model-free system is cognitively inexpensive and fast (but inflexible) and is thought to underlie habitual control. To better understand this balance in obesity, the current study investigates relative reliance on model-based and model-free control of choice behaviour.

Indirect evidence links obesity to reduced model-based, or rather, goal-directed control. Previous outcome devaluation studies tapping into goal-directed and habitual control of food choices in obesity have shown a negative correlation between goal-directed control and degree of obesity in humans^13,14^. That is, the higher the BMI, the less participants adjusted their food choices after devaluation of one of the two choices. Behavioural adjustment after outcome devaluation of non-food rewards related positively to model-based, but not model-free control, in healthy human participants performing a two-step decision-making task^15–17^ (but see^18^). Alterations in model-based vs. model-free control have been associated with behavioural inflexibility as observed in clinical populations such as metamphetamine addiction, obsessive compulsive disorder, and binge eating disorder^19,20^, as well as in a general population sample reporting symptoms of the same disorders and of other eating disorders^21^. However, Voon et al.^19^ did not find differences in model-based and model-free control between obese participants without binge eating disorder and non-obese control participants. The absence of an association between obesity and model-based or model-free control seems surprising, given the above-mentioned obesity-related performance differences in simple reinforcement learning tasks and outcome devaluation tasks, resembling more model-free and model-based control, respectively.

We propose two reasons why the study by Voon et al.^19^ might have lacked power to detect obesity-related group differences in model-based and model-free control. First, rather subtle behavioural alterations are to be expected in obese individuals that are physically healthy. With a relatively low contrast in body mass index (BMI) between the obese and non-obese group (BMI [kg/m^2^]: obese: M = 31.49, SD = 3.6; non-obese: M = 23.54, SD = 2.9), and an average BMI for the obese group only slightly above the cutoff for obesity (> 30 kg/m^2^), such behavioural alterations may be difficult to detect. Second, the relationship between BMI and model-based and model-free control may in fact be quadratic in nature, thus masking potential obesity-related differences. A quadratic relationship with degree of obesity has indeed been observed for reward sensitivity^22^ and cognitive restraint of eating behaviour^23^. Furthermore, obesity may quadratically relate to alterations in striatal dopamine tone^24^. This is relevant because there is accumulating evidence that different measures and manipulations of dopamine transmission overall relate positively to model-based control as measured in the two-step task^25–29^.

In the current study, we aimed to address the two issues raised above by including (1) more highly obese individuals to boost the contrast between groups, and (2) an intermediate overweight group for more sensitivity to detect the existence of potential linear or quadratic relationships between weight status and behavioural control. The original two-step task was implemented to disentangle and directly compare the reliance on model-based and model-free control^16,25,30^. We hypothesized that weight status relates to the degree to which individuals rely on model-based and model-free learning, and that it may do so in a linear or quadratic manner.

## Materials and methods

### Participants

The results reported in this study are based on data from 90 healthy right-handed participants in a wide BMI range (45 women; age [years]: M = 26.9; SD = 3.6; range 21–35; BMI [kg/m^2^]: M = 27.9, SD = 6.4, range 18.4–47.6). Participants were recruited based on their BMI status, i.e., normal-weight [n(women) = 31(16), BMI [kg/m^2^] = 18.5–24.9], overweight [n(women) = 29(14), BMI [kg/m^2^] = 25–29.9] and obese [n(women) = 30(15), BMI > 30] (Table 1). Note that the reported data were acquired in two parts. Fifty-seven datasets were acquired as a part of several studies running in the department between October 2012 and August 2014. Data acquisition of overweight and obese participants was not completed at the time due to logistic reasons. To finally conclude the study, the remaining participants were tested between February and March 2018 (n = 37, for details see Supplemental Figure S1). Part of the reported data have previously been published in a study comparing relative reliance on model-based and model-free control to habit propensity in a slips-of-action task in specifically normal-weight women and men (n = 28)^16^. Participants were tested at the Department of Neurology of the Max Planck Institute for Human Cognitive and Brain Sciences (Leipzig, Germany) and received monetary compensation on an hourly basis, as well as a bonus based on their task performance (between 3 and 10€; *M* = 6.5€, *SD* = 0.82). All participants gave written consent prior to the study. The study was carried out in accordance with the Declaration of Helsinki and approved by the Ethics Committee at the University of Leipzig, Germany.

**Table 1:** Group characteristics displaying mean (standard deviation) and range if not otherwise stated, followed by the test-statistic and p-value of group comparison for each measure.

	Normal-weight		Overweight		Obese		*p*	*Test-statistic*
n	31		29		30			
sex (F:M)	16:15		14:15		15:15		ns	0.07^b^
Age	26.9 (3.3)	21–34	26.0 (3.7)	21–35	27.8 (3.8)	22–34	0.166	1.8^c^
BMI (kg/m^2^)	21.6 (1.8)	18.4–24.8	26.9 (1.3)	25.1–29.9	35.4 (4.5)	30.2–47.6	< 0.001	79.1^d^
**Cognitive tests**								
Non-verbal IQ^a^	119.9 (12.1)	95.0–136.5	117.3 (10.6)	93.0–136.5	111.2 (16.4)	85.0–136.5	0.084	5.0^d^
VPA score	12 (3.9)	3–18	13.2 (3.1)	7–18	12.2 (3.2)	6–18	0.381	1.0^c^
**Self-report questionnaires**								
BDI	3.6 (3.3)	0–14	4.9 (3.2)	0–11	6.8 (4.2)	0–17	0.003	11.5^d^
**BIS/BAS**								
BIS	20.0 (3.4)	14–28	19.8 (4.3)	11–27	19.5 (4.0)	7–27	0.884	0.1^c^
BAS drive	12.1 (2.1)	7–16	12.0 (1.8)	9–16	11.5 (1.7)	8–16	0.449	0.8^c^
BAS fun	12.1 (1.8)	9–16	12.0 (1.8)	8–15	12.0 (1.9)	8–16	0.972	0.03^c^
BAS reward	16.8 (2.0)	12–20	17.0 (2.1)	11–20	16.0 (2.0)	10–19	0.141	2.0^c^
**TFEQ**								
Restraint	5.0 (3.2)	0–15	8.1 (4.6)	0–18	6.4 (4.7)	0–18	0.027	7.2^d^
Disinhibition	4.9 (2.1)	0–9	6.3 (3.3)	2–15	8.3 (3.3)	3–16	< 0.001	16.9^d^
Hunger	5.7 (3.3)	1–13	5.1 (4.1)	0–13	7.1 (3.4)	1–14	0.066	5.4^d^
**UPPS**								
Urgency	26.8 (5.8)	15–42	25.4 (5.1)	17–36	27.7 (6.6)	13–39	0.314	1.2^c^
(lack of) Premeditation	22.2 (4.1)	12–31	22.7 (4.6)	16–36	22.3 (4.0)	12–29	0.904	0.1^c^
(lack of) Perseverance	20.1 (6.0)	12–44	19.4 (5.6)	10–34	21.3 (5.3)	12–34	0.438	0.8^c^
Sensation seeking	31.8 (6.6)	18–44	31.6 (8.5)	17–48	28.0 (7.4)	14–40	0.090	2.5^c^
YFAS (#symptoms)	0.8 (0.7)	0–2	1.3 (1.4)	0–7	1.9 (1.0)	0–4	< 0.001	17.3^d^

*N* number of participants; *F:M* the ratio of females to males; *VPA* visual paired associates test of the Wechsler Memory Scale; *BDI* Beck’s Depression Inventory; *BIS/BAS* Behavioural Inhibition System/Behavioural Activation System; *TFEQ* Three-Factor Eating Questionnaire; *UPPS* Urgency, lack of Premeditation, lack of Perseverance, and Sensation seeking; *YFAS* Yale Food Addiction Scale.

^a^Non-verbal IQ was calculated based on the Viennese Matrices Test (VMT).

^b^Chi square test for frequency data (degrees of freedom: 2).

^c^F-test with for normally distributed scores (degrees of freedom: 2.87).

^d^Independent-Samples Kruskal–Wallis Test of distributions for non-normally distributed scores (degrees of freedom: 2).

After having provided informed consent, weight and height of the participants was measured, followed by the two-step task (for details see “Experimental paradigm”). Participants were then asked to complete a number of self-report questionnaires—validated in German—for characterizing the sample: Beck’s Depression Inventory (BDI)^31^ to assess possible depressive symptoms (cut-off for exclusion > 18, indicating possibility of moderate to severe depression), the Behavioural Inhibition System/Behavioural Activation System questionnaire (BIS/BAS)^32,33^ to assess punishment and reward sensitivity, the Three-Factor Eating Questionnaire (TFEQ)^34,35^ to assess eating behaviour in terms of cognitive restraint, disinhibition and hunger, the UPPS Impulsive Behaviour Scale^36,37^ to assess impulsive behaviour in terms of Urgency, lack of Premeditation, lack of Perseverance, and Sensation seeking, and the Yale Food Addiction Scale (YFAS)^38,39^ to assess symptoms that could be indicative of food addiction. Finally, participants performed several cognitive tests to examine their potential relation to performance on the task: the Viennese Matrices Test (VMT)^40^ to assess non-verbal IQ. We also administered a computerized version of the Visual Paired Associates test of the Wechsler Memory Scale (VPA)^41,42^ to assess visual short term memory. Participants were included if none of the following exclusion criteria applied: estimated non-verbal IQ (< 85 based on the VMT), known metabolic disorders (e.g., diabetes), smoking, (history of) neurological, psychiatric, or eating disorders, symptoms of depression, drug or alcohol dependence, current pregnancy, and psychological treatment. In total 94 participants were tested of which three participants did not complete the experimental paradigm and one participant was excluded from analysis because of an estimated non-verbal IQ below 85.

### Experimental paradigm

**Figure 1:**
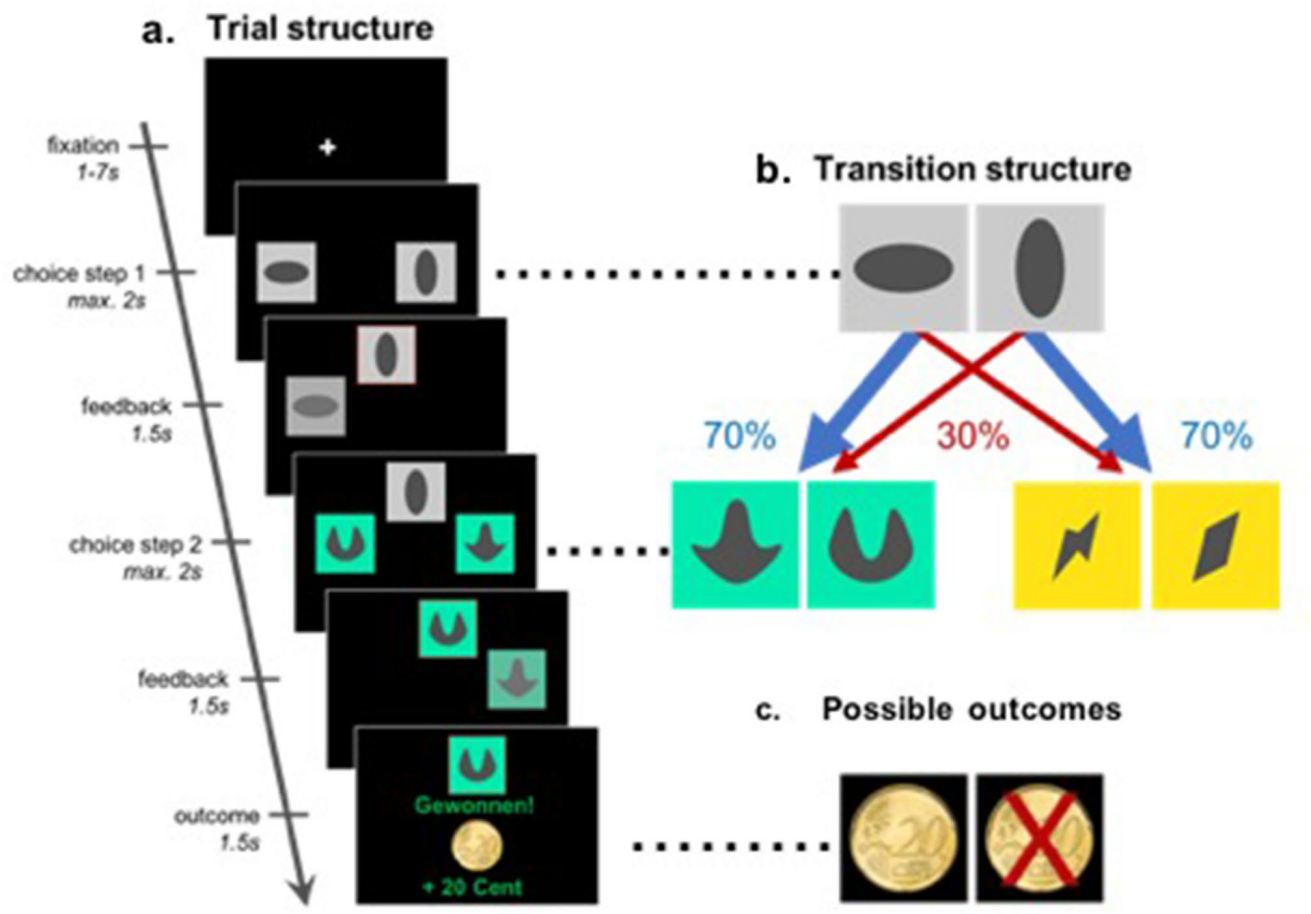
The two-step task^25,30^. (**a**) Trial structure of an example trial with a rare transition, which allows for the dissociation of model-based and model-free control of behaviour. (**b**) Transition structure showing how each first-stage stimulus (grey) leads to one of the two second-stage stimulus pairs (green or yellow) in 70% of the trials (common, blue arrows) and to the other pair in 30% of the trials (rare, red arrows). (**c**) Possible outcomes (reward, no reward). Reward probability for the four second-stage stimuli varies throughout the task according to random walks to encourage continuous learning.

We administered a sequential decision making task^16,25,30^, in which participants were asked to make two subsequent decisions on each trial to earn a monetary reward (20 cents) or no reward (Fig. 1a). At the first stage, participants were asked to choose between two grey stimuli, which would bring them to one of two second-stage stimulus pairs (the green or yellow pair). One of the grey first-stage stimuli was connected commonly (70%) to the green and rarely (30%) to the yellow stimulus pair, and vice versa for the other grey stimulus (Fig. 1b). The first-stage stimuli and transition probabilities were fixed throughout the experiment. After selecting one of the two second-stage stimuli, participants either received the monetary reward or not (Fig. 1c). The probability of receiving reward for each of the four second-stage stimuli changed slowly and continuously according to Gaussian random walks to ensure continuous learning. The changes were kept consistent for all participants performing the experiment. Participants completed a total of 201 trials. Prior to the experiment, participants went through elaborate computer-based instructions and were then asked to explain the task including its first-stage transition probabilities to the experimenter. Open questions were addressed by the experimenter. The instructions included a detailed knowledge of common (70%) and rare (30%) transitions after first-stage choices, and the slowly changing probabilities after second-stage choices. After the instructions participants performed 56 training trials with a different set of stimuli. Participants were made aware that the height of their financial bonus depended on the accumulated reward in the task. The bonus was based on a randomly drawn subset of trials.

### Data analysis

Calculation of first-stage stay probabilities on the two-step task, as well as computational modeling of participants’ choice behaviour were performed using in-house scripts in Matlab (version 2017b, The MathWorks, Inc.). Statistical analyses of self-reported, behavioural, and computational data were run in R Studio (version 3.4.4., R Core Team, 2018^43^) and SPSS (version 24, IBM Corp., 2018). The R package ggplot2 was used to plot the results^44^.

Shapiro–Wilk’s test of normality and Levene’s test of equality of variance were ran for all group characteristics, including scores on self-reported questionnaires and neuropsychological tests, as well as for the accumulated reward (i.e., number of rewarded trials), raw stay probabilities (per condition), reaction times, and for the estimated model parameters.

The alpha level was set to 0.05 (α = 0.05) for all a priori analyses of interest. Note that for post hoc analyses, we did not correct for multiple comparisons as these results are exploratory and should be interpreted as such.

Partial *η*^2^ (*η*_*p*_^2^) is reported as an effect size for all parametric univariate analyses because it meaningfully describes effects in a design in which multiple measures have been experimentally manipulated (as in the two-step task), and it yields very similar estimates as *η*^2^ for analyses that only include a between-group variable^45,46^. Note that *η*_*p*_^2^ does not depend on the number of variables in the model and, thus, can be compared across studies. For non-parametric Kruskal–Wallis tests, *η*^2^_H_ was calculated as follows: (H − k + 1)/(n − k), with *H* reflecting the test statistic, *k* the number of groups, and *n* the total sample size^47^.

To check the robustness of our findings and rule out that any observed effect of group on behaviour could have been driven by age^21,48,49^ or IQ^16,21,50,51^ rather than weight status, we reran all models post hoc including age and non-verbal IQ as covariates of no interest.

#### Characterization of the groups

We tested for group differences in age and sex to confirm that the groups were well-matched. BMI was analysed to confirm the grouping of participants into normal-weight, overweight and obese participants. Group analysis of cognitive tests (including non-verbal IQ) and self-reported questionnaire data were run to further characterize the sample.

For normally distributed data (age, VPA score, BIS/BAS, UPPS), we ran a one-way ANOVA with between-subjects factor weight group for each measure. Upon violation of the assumption of normality or equality of variance (BMI, non-verbal IQ, BDI, TFEQ, YFAS symptom score), the Kruskal–Wallis test by ranks was performed. Sex distribution between groups was analysed using Chi-Square Test. Group differences were followed up by post hoc parametric (independent T-test) or nonparametric (Mann–Whitney U Test) pairwise comparisons.

#### Raw behaviour according to first-stage stay probabilities

Investigating the likelihood with which participants choose a first-stage stimulus depending on the previous trial type (Rewarded/Unrewarded, Common/Rare), gives an insight into how much they relied on model-based or model-free control. Therefore, we calculated first-stage stay probabilities as the proportion of trials in which participants chose the same first-stage stimulus as in the previous trial (coded as ‘stay’) for each of the conditions (Rewarded Common, Rewarded Rare, Unrewarded Common, Unrewarded Rare). We then analysed participants’ stay probabilities using ANOVA with the between-subject factor Group (Normal-weight, Overweight, Obese), and within-subject factors Reward (Rewarded, Unrewarded) and Transition (Common, Rare). Because the aim was to test for a three-way interaction and the group sizes are well balanced, type III sums of squares were calculated in this analysis.

A purely model-free agent relies on whether or not the previous trial was rewarded, irrespective of transition probability (Common/Rare). If rewarded, the previous first-stage choice should be repeated. If not, it may be better for the model-free agent to switch to the other first-stage stimulus. As a consequence, model-free control is reflected in a main effect of Reward. On the other hand, a purely model-based agent optimally relies both on reward and transition probability of the previous trial. A model-based agent will also stay with a previous first-stage choice when a common trial was rewarded, and switch when a common trial was not rewarded. However, the model-based agent differs in choice behaviour following rare trials. That is, in contrast to a purely model-free agent, a model-based agent can infer that when a rare trial was rewarded, reward probability on the current trial is higher if one chooses the other first-stage stimulus (switch), and vice versa for unrewarded rare trials (stay). Model-based control is therefore reflected in the interaction between Reward and Transition. Here, we were mainly interested in group differences in model-based and model-free control and thus focused on the Group × Reward × Transition interaction and Group × Reward interaction on stay probabilities, respectively.

We hypothesized that the relationship between weight status and model-based or model-free control might be linear or quadratic in nature. To investigate the nature of these relationships, we next performed planned pairwise group comparisons on the Reward × Transition interaction term [i.e., (Rewarded Common − Rewarded Rare)  −  (Unrewarded Common − Unrewarded Rare)] and on the main effect of Reward [i.e., (Rewarded Common + Rewarded Rare) − (Unrewarded Common + Unrewarded Rare)] on stay probabilities.

Finally, we ran two post hoc linear models (lm() from the R stats package): (1) on the Reward × Transition interaction term, and (2) on the main effect of Reward to investigate the existence of a linear and quadratic relationship with BMI on a continuous scale. Both models included BMI and BMI^2^ as orthogonal predictors.

#### Computational modeling

To investigate how participants’ choices were affected by reward and transition probability throughout the experiment rather than in the previous trial alone, we computationally modeled choice behaviour. We implemented a hybrid of a model-free and model-based reinforcement algorithm as is described in detail in our previous work^16,25^ and in the original paper^30^.

In short, the model-free algorithm (SARSA(λ)) included a learning rate for each stage (α_1_, α_2_) and a parameter λ, which allows the second stage prediction error to affect the next first-stage values (Q). The model-based algorithm learns values by planning forward and computes first-stage values by multiplying the value of the better second-stage option with the associated transition probabilities. Then, the model-free and model-based first-stage decision values are connected in the hybrid algorithm:

$$Q_{net}\left({s}_{A},{a}_{j}\right)=\omega { Q}_{MB}\left({s}_{A},{a}_{j}\right)+(1- \omega ){ Q}_{MF}\left({s}_{A},{a}_{j}\right)$$

where $Q_{net}\left({s}_{A},{a}_{j}\right)$ denotes the decision value of the chosen stimulus ${a}_{j}$ from the first stage stimulus pair ${s}_{A}$, and $\omega$ captures the relative weighting of the model-based (${Q}_{MB}\left({s}_{A},{a}_{j}\right)$) and model-free algorithm (${Q}_{MF}\left({s}_{A},{a}_{j}\right)$). The weighting parameter $\omega$ is the main parameter of interest and can take a value between 0 and 1. If $\omega$ = 1, first-stage choices are purely controlled by model-based control, and if $\omega$ = 0, they are purely controlled by model-free control. Note that at the second stage $Q_{net}={ Q}_{MB}\,{=Q}_{MF}$.

Finally, the decision values were transformed into action probabilities using the softmax function for $Qnet$:

$$P(a_{i,t} = a{|}s_{i,t} {)} = \frac{{exp\left( {\beta_{i} \left[ { Q_{net} \left( {s_{i,t} ,a} \right) + \rho \cdot rep\left( a \right)} \right]} \right)}}{{\sum\limits_{{a^{\prime}}} {{\text{exp}}\left( {\beta_{i} \left[ { Q_{net} \left( {s_{i,t} ,a^{\prime}} \right) + \rho \cdot rep\left( {a^{\prime}} \right)} \right]} \right.} }}$$

where ${\beta }_{i}$ controls the stochasticity of choices at stage $i$ = 1 or 2, and repetition parameter $\rho$ reflects choice perseveration at the first stage.

The model had a total of seven parameters that were bounded by transforming them to a logistic $({\alpha }_{1},{\alpha }_{2}, \lambda , \omega )$ or exponential $({\beta }_{1},{\beta }_{2})$ distribution. To infer the maximum-a-posteriori estimate of each parameter for each subject, the (empirical) Gaussian prior distribution was set to the maximum-likelihood estimates given the data of all participants and then expectation–maximization was used^52^. We report the negative log-likelihood (− LL) as a measure of model fit.

We assessed group differences in $\omega$ using ANOVA with between-group factor weight status. Planned pairwise comparisons were performed as part of the ANOVA or using Mann–Whitney U test as a nonparametric alternative. For each of these analyses, the alpha level was set at 0.05. Finally, we investigated the relationship between $\omega$ and weight status on a continuous scale by running a post hoc linear regression model including BMI and BMI^2^ as orthogonal predictors.

After having detected between-group differences on the model parameters’ of interest, an important sanity check is whether the inferred parameters actually reproduce the observed behavioural data in terms of stay probabilities. To do so, we re-ran the model based on each individual’s inferred parameters to generate data for each individual (1000 simulations per subject) and performed the original ANOVA.

We then ran simulation recovery analyses for the model to assess whether the model parameters captured the observed behavioural data. Based on the estimated parameters, we simulated choice behaviour on the task and investigated stay probabilities. The reported significant Group × Reward × Transition interaction was fully reproduced indicating that the model captured important aspects of the data (Supplemental Figure S2).

Finally, to confirm that the chosen hybrid model including $\lambda$ was the best-fitting algorithm in this study, we compared the model to less complex models. To avoid inclusion of numerous combinations of parameters, we focus on models that capture distinct behaviour in this task by setting $\omega$ to 1 or 0, and $\lambda$ to 0 or fitting it as a free parameter. This gives four additional models: (1) a hybrid model without $\lambda$ ($\omega$ = 0), (2) a model only including the model-based learning algorithm ($\omega$ = 1, $\lambda$ can not be fitted), (3) a model only including the model-free learning algorithm with $\lambda$ ($\omega$ = 0), and (4) the same model-free model without $\lambda (\omega = 0)$. Integrated Bayesian Information Criterion (BIC) is reported for all models^52^.

## Results

### Characterization of the groups

Table 1 summarizes the weight groups [normal-weight (NW), overweight (OW), and obese (OB)] in terms of age, sex and BMI, as well as in terms of their scores on the cognitive tests and self-report questionnaires. The groups were well matched on sex and age, and did not differ in visual short-term memory (VPA), or non-verbal IQ as measured on the Viennese Matrices Test (VMT). However, a trend-level group difference was observed for non-verbal IQ, with numerically higher IQ scores for the normal-weight and overweight relative to the obese group (Table 1). We did observe a group difference in the average number of depressive symptoms (*KW*(2) = 11.5, *p* = 0.003, *η*^2^_H_ = 0.11) even though the scores are not clinically relevant in the current sample. This difference was driven by the obese participants having a higher symptom score relative to normal-weight, but not overweight, participants (post hoc pairwise comparisons: NW vs. OB, *p* = 0.004; OW vs. OB, *p* = 0.137; NW vs OW, *p* = 0.254). Post hoc covariate analyses of behavioural and computational data controlling for BDI score did not change the primary effects of interest (see “Supplemental Materials” for statistics). The average number of food addiction symptoms also differed between the groups (KW(2) = 17.3, *p* < 0.001, *η*^2^_H_ = 0.18), again, driven by a higher number of symptoms for obese relative to normal-weight, but not overweight, participants (post hoc pairwise comparisons: NW vs. OB, *p* < 0.001; OW vs. OB, *p* = 0.159; NW vs OW, *p* = 0.242). In terms of self-reported eating behaviour (TFEQ) the groups differed in disinhibition (KW(2) = 16.9, *p* < 0.001, *η*^2^_H_ = 0.17) and restraint (KW(2) = 7.2, *p* = 0.027, *η*^2^_H_ = 0.06). Disinhibition scores were higher for obese relative to both normal-weight and overweight participants and somewhat higher for overweight relative to normal-weight participants (post hoc pairwise comparisons: NW vs. OB, *p* < 0.001; OW vs. OB, *p* = 0.010; NW vs OW, *p* = 0.076). Restraint scores were highest for overweight participants and lower for normal-weight, but not obese participants (post hoc pairwise comparisons: NW vs. OB, *p* < 0.375; OW vs. OB, *p* = 0.374; NW vs OW, *p* = 0.013). No other group differences were observed.

### Raw behaviour according to first-stage stay probabilities

**Figure 2:**
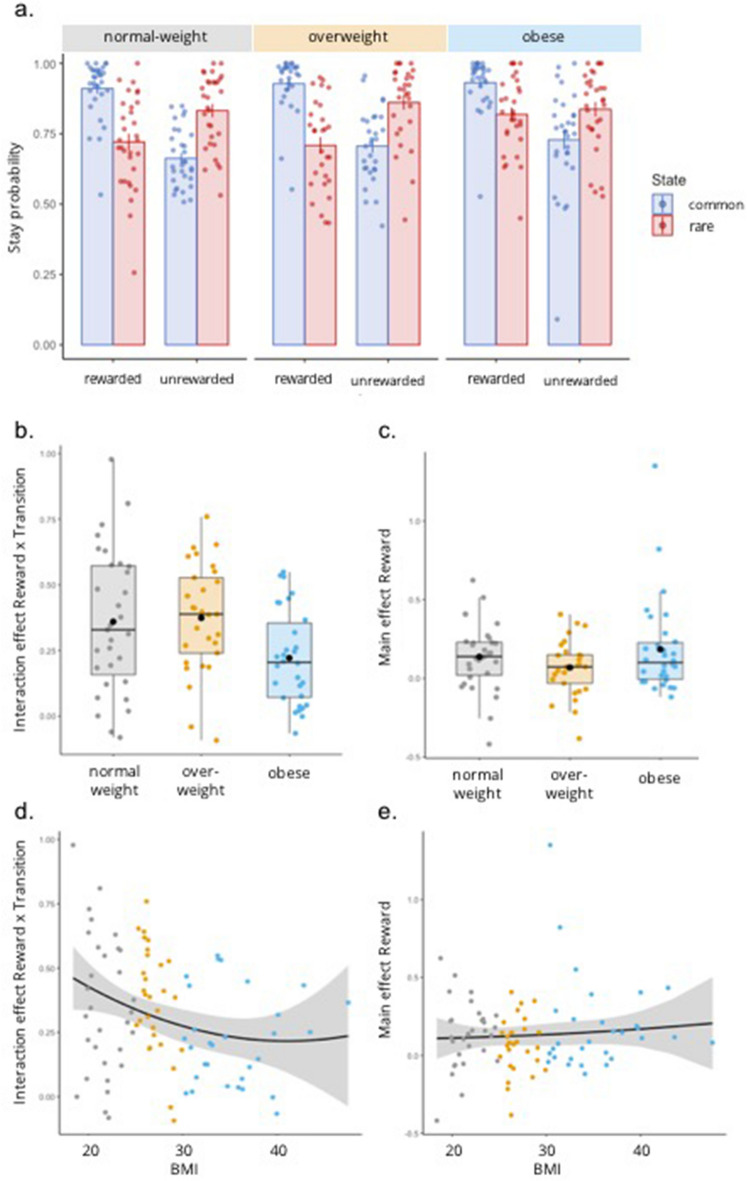
Stay probabilities. (**a**) Average stay probabilities per condition for each group. Error bars represent ± 1 SEM. (**b**) On the group level, the use of a model-based choice strategy (i.e., the Reward × Transition interaction term) was lower for obese relative to normal-weight and overweight participants, whereas (**c**) the use of a model-free choice strategy (i.e., the main effect of Reward) did not differ significantly between groups. The box plots in (**b**) and (**c**) show the median and interquartile range for each group, with the black dot denoting the mean. (**d**) On the continuous level, the Reward × Transition interaction term was negatively related to BMI, with no additional significant quadratic relationship. (**e**) No linear or quadratic relationship was observed between BMI and the main effect of Reward. The scatter plots in (**d**) and (**e**) show the model fit (black line) and confidence interval (shaded) of the respective regression models with predictors BMI and BMI^2^. Individual data points are color-coded based on weight group for illustrative purpose.

Analysis of stay probabilities (Fig. 2a) revealed that participants’ first-stage choices were significantly affected by reward (main effect Reward: F(1,87) = 27.2, *p* < 0.001, *η*_*p*_^*2*^ = 0.238) as well as by the combination of reward and transition probability (interaction Reward × Transition: F(1,87) = 183.4, *p* < 0.001, *η*_*p*_^*2*^ = 0.678) on the previous trial. This is in line with previous research^25,30^ and suggests that, across groups, the participants relied on both model-based and model-free choice strategies, respectively. Transition probability alone did not significantly affect participants’ first-stage choices (Transition: F(1,87) = 3.4, *p* = 0.070, *η*_*p*_^*2*^ = 0.037).

The weight groups significantly differed in the use of a model-based choice strategy (Fig. 2b) as reflected by a significant three-way Group × Reward × Transition interaction on stay probabilities (*F* (2,87) = 4.3, *p* = 0.017, *η*_*p*_^*2*^ = 0.090), but not in the use of a model-free choice strategy (Group × Reward: *F* (2,87) = 1.8, *p* = 0.174, *η*_*p*_^*2*^ = 0.039, Fig. 2c). Planned comparisons of the Reward × Transition interaction between groups showed that the three-way interaction was driven by a significantly higher interaction term for normal-weight relative to obese (*p* = 0.017) and for overweight relative to obese (*p* = 0.010) participants, whereas normal-weight and overweight participants did not differ from each other (*p* = 0.817).

We observed no Group × Transition interaction (*F* (2,87) = 1.2, *p* = 0.297, *η*_*p*_^*2*^ = 0.028), nor a main effect of Group (*F* (2,87) = 1.7, *p* = 0.187, *η*_*p*_^*2*^ = 0.038) on stay probabilities. These results suggest that choices of obese participants relied relatively less on model-based control than those of normal-weight and overweight participants.

Post hoc simple effects analyses were performed to further investigate the three-way interaction on stay probabilities and revealed a striking difference between the groups. Interestingly, we observed a Group × Reward interaction for rare (*F*(2,87) = 4.2, *p* = 0.018), but not common trials (*F*(2,87) < 1, *p* = 0.497). This in turn was driven by a simple main effect of Group on stay probabilities following rewarded rare trials (*F*(2,87) = 4.6, *p* = 0.012), but not unrewarded rare trials (*F*(2,87) < 1, *p* = 0.688). The simple effect of Group was also reflected in a Group × Transition interaction for rewarded (*F* (2,87) = 3.8, *p* = 0.026), but not unrewarded trials (*F* (2,87) = 2.4, *p* = 0.100). Finally, pairwise group comparisons of rewarded rare trials showed that obese participants were more likely to stay with their previous first-stage choices when a rare trial had been rewarded relative to normal-weight (*t*(59) = − 2.5, *p* = 0.014) and overweight participants (*t*(57) = − 2.9, *p* = 0.006), with no difference between normal-weight and overweight participants (*t*(58) = 0.3, *p* = 0.766). This is of interest because it is participants’ behaviour following rare trials that allows us to dissociate model-based from model-free control. Increased staying after a rare rewarded trial hints at more model-free control, even though this effect was not sufficiently strong to come out as a significant interaction between Group and Reward. Nevertheless, it seems that the observed group difference in model-based control may in fact be driven by enhanced reliance on model-free computations (see “Discussion” for more).

Another means of probing the model-based control system in the framework of this task, is to investigate second-stage reaction times^25,53^. Since a model-based agent uses knowledge of the likelihood of the transition into a second-stage state, encountering an unexpected rare rather than an expected common transition should increase reaction times for choices at the second stage. In a post hoc analysis, we indeed observed a main effect of Transition on second-stage reaction times, which reflected significantly larger reaction times following a rare relative to a common transition (*M(SD)*_rare_ = 992.3 (137.5) ms > *M(SD)*_common_ = 731.1 (101.2) ms, *F* (1,87) = 366.2, *p* < 0.001, *η*_*p*_^*2*^ = 0.808). We did not, however, observe a significant Group by Transition interaction (*F* (2,87) < 1, *p* = 0.411, *η*_*p*_^*2*^ = 0.020), which is in line with the above interpretation that the observed Group × Reward × Transition interaction on stay probabilities may not purely reflect a group difference in model-based control*.* The absence of the Group by Transition interaction could not be explained by general reaction times differences between the groups, as the groups did not differ in their RTs overall for either stage 1 (*M(SD)* = 656.7 (8.8) ms, *F* (2,87) < 1, *p* = 0.847, *η*_*p*_^*2*^ = 0.004), or stage 2 decisions (*M(SD)* = 808.3 (9.6) ms, *F* (2,87) < 1, *p* = 0.808, *η*_*p*_^*2*^ = 0.005).

Next, we addressed the question if reliance on model-based and model-free control related to obesity in a linear and/or quadratic manner. Because the traditional weight categories of normal-weight, overweight and obese individuals reflect unequal intervals in terms of BMI, we turned to BMI as a continuous variable, even though the study was designed for group-based analyses. We ran two linear regression models including BMI and BMI^2^ as orthogonal predictors in each, and investigated their relationship with the (1) Reward × Transition interaction term, and (2) the main effect of Reward on stay probabilities. BMI related negatively to the Reward × Transition interaction term (*β*_*BMI*_ = − 0.28, *p* = 0.007), but no additional quadratic relationship was observed (*β*_*BMI*_^*2*^ = 0.10, *p* = 0.319) (Fig. 2d). Together, BMI and BMI^2^ explained a significant proportion of variance in the effect of Reward and Transition on choice strategy (adjusted *R*^*2*^ = 0.069, *F*(2,87) = 4.3, *p* = 0.017). In line with the absence of a Group × Reward effect on stay probabilities, we did not observe a linear or quadratic relationship between BMI and the main effect of Reward on stay probabilities (*β*_*BMI*_ = 0.08, *p* = 0.463; *β*_*BMI*_^*2*^ = 0.01, *p* = 0.892) (Fig. 2e), nor did the model explain a significant proportion of variance (adjusted *R*^*2*^ = − 0.016, *F* (2,87) = 0.3, *p* = 0.756). Note that a post hoc analysis results suggest that the linear relationship between BMI and the Reward × Transition interaction term may be moderated by BDI score (see “Supplemental Materials” for statistics).

### Accumulated reward

The accumulated reward (i.e., sum of rewarded trials) of participants was analyzed as a measure of overall performance. The groups did not differ in the sum of rewarded trials throughout the experiment (*M* = 97.2, *SD* = 7.7, *F* (2,87) = 1.6, *p* = 0.209, *η*_*p*_^*2*^ = 0.035). The sum of rewarded trials also did not correlate to participants’ tendency to rely on model-based or model-free choice strategies in any of the measures of interest (*p*’s > 0.299).

### Computational modeling of choice behaviour

Computational modeling of behaviour allowed us to take into account participants’ choices throughout the experiment rather than only considering the effect of the previous trial. For a summary of all parameters and group comparisons, see Table 2.

**Table 2:** Summary and group comparisons of all model parameters.

			25%	50%	75%		
ω	NW	0.66 (0.09)	0.58	0.68	0.72	5.3^a^	0.007
	OW	0.68 (0.09)	0.63	0.68	0.75		
	OB	0.60 (0.11)	0.52	0.60	0.70		
α_1_	NW	0.47 (0.17)	0.33	0.48	0.63	< 1^b^	0.867
	OW	0.47 (0.21)	0.34	0.55	0.65		
	OB	0.46 (0.29)	0.23	0.42	0.73		
α_2_	NW	0.54 (0.23)	0.41	0.62	0.70	< 1^a^	0.560
	OW	0.56 (0.19)	0.47	0.56	0.68		
	OB	0.50 (0.21)	0.35	0.50	0.70		
β_1_	NW	7.7 (2.6)	5.3	7.8	9.0	1.6^b^	0.447
	OW	9.0 (4.0)	6.3	8.1	10.7		
	OB	8.6 (3.2)	6.9	7.6	11.3		
β_2_	NW	4.3 (1.7)	2.8	4.1	5.4	< 1^b^	0.955
	OW	4.0 (1.2)	3.2	4.2	4.8		
	OB	5.2 (3.7)	3.3	4.0	5.4		
λ	NW	0.53 (0.23)	0.36	0.54	0.71	< 1^a^	0.967
	OW	0.53 (0.18)	0.38	0.56	0.69		
	OB	0.55 (0.22)	0.41	0.55	0.69		
ρ	NW	0.14 (0.05)	0.10	0.14	0.18	3.0^a^^,c^	0.057
	OW	0.14 (0.04)	0.11	0.13	0.16		
	OB	0.15 (0.06)	0.13	0.16	0.18		
-LL	NW	175.7 (40.9)	141.1	174.0	207.6	1.5^a^	0.225
	OW	169.8 (44.8)	137.8	157.4	188.6		
	OB	155.7 (50.7)	128.3	157.9	193.3		

*NW* normal-weight, *OW* overweight, *OB* obese.

^a^F-test for normally distributed parameters (degrees of freedom: 2.87).

^b^Independent-Samples Kruskal–Wallis Test of distributions for non-normally distributed parameters (degrees of freedom: 2).

^c^One formal outlier was observed in the obese group and excluded from the analysis of this parameter (degrees of freedom: 2.86).

**Figure 3:**
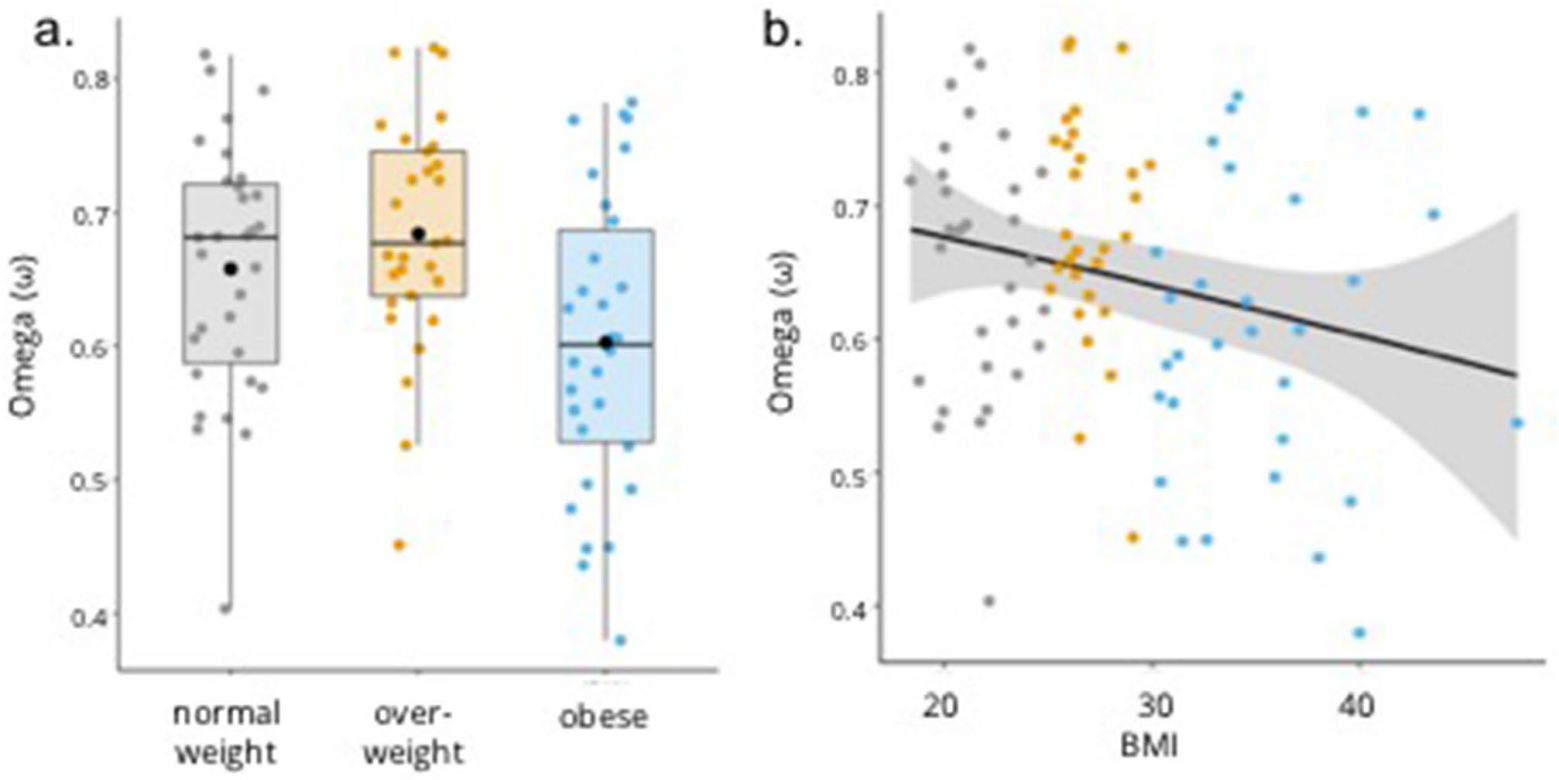
Relative reliance on model-based and model-free control (omega). (**a**) On the group level, omega was significantly lower for obese relative to normal-weight and overweight participants. The box plot reflects the median, interquartile range, and mean value (black dot) for each weight group. (**b**) On the continuous level, omega was negatively related to BMI, with no additional significant quadratic relationship. The scatter plot shows the model fit (black line) and confidence interval (shaded) of the regression model. Individual data points are color-coded based on weight group for illustrative purposes.

The parameter *ω* was of initial interest because it reflects participants’ relative reliance on model-based vs. model-free control. A purely model-based agent has an *ω* of 1, whereas a purely model-free agent has an *ω* of 0*.* As expected, we observed a significant group effect on *ω* (*F* (2,87) = 5.3, *p* = 0.007, *η*_*p*_^*2*^ = 0.109) (Fig. 3a). Planned comparisons showed that the group effect on *ω* was driven by higher values for normal-weight relative to obese (*t*(59) = 2.1, *p* = 0.042) and overweight relative to obese participants (*t*(57) = 3.1, *p* = 0.003). Although overweight participants numerically had the highest *ω* values, there was no statistical difference with normal-weight participants (*t*(58) = -1.1, *p* = 0.265).

To investigate the nature of the relationship between *ω* and weight on a continuous scale (i.e., BMI)*,* we again ran a post hoc regression model including the linear term BMI and quadratic term BMI^2^ as predictors. The linear term related negatively to values of *ω* with lower values in individuals with a higher BMI (*β*_*BMI*_ = − 0.23, *p* = 0.030), whereas the quadratic term did not significantly add to the model (*β*_*BMI*_^*2*^ = − 0.005, *p* = 0.964) (Fig. 3b). In total, the model explained 3.1% of variance in *ω* (adjusted R^2^ = 0.031, *F* (2,87) = 2.4, p = 0.093), which reflects only a small effect of BMI on reliance on model-based vs. model-free control. Similar to post hoc analysis of the relationship between BMI and the interaction effect of Reward × Transition on stay probabilities, the negative relationship between BMI and *ω* may be moderated by BDI score (see “Supplemental Materials” for statistics).

None of the other model parameters differed significantly between the groups (Table 2). This indicates that the groups did not differ in terms of first or second stage learning rates (α_1,_ α_2_), stochasticity of first or second stage choices (β_1,_ β_2_), the tendency to persevere independent of reward or transition (ρ), the eligibility parameter (λ), and importantly, how well the model fit participants’ data (− LL).

Finally, to confirm that the chosen hybrid model including $\lambda$ was the best-fitting algorithm in this study, we compared the model to four less complex models by setting $\omega$ to 1 or 0, and $\lambda$ to 0 or fitting it as a free parameter. Comparing the Bayesian Information Criterion (BIC) scores of the five models across the entire sample as well as in each group separately shows clear superiority for the ‘full’ hybrid model in each case (Table 3).

**Table 3:** Model comparison.

	Hybrid with $\lambda$	Hybrid without $\lambda$	MB	MF with $\lambda$	MF without $\lambda$
All (n = 90)	**32,140.72**	+ 273.55	+ 402.40	+ 1089.91	+ 1733.09
NW (n = 31)	**11,492.61**	+ 122.75	+ 112.43	+ 423.32	+ 689.88
OW (n = 29)	**10,696.63**	+ 62.30	+ 85.73	+ 453.02	+ 630.55
OB (n = 30)	**10,075.42**	+ 70.79	+ 151.12	+ 195.87	+ 377.26

Values reflect Bayesian Information Criterion (BIC) scores.

Values in bold reflect the winning model when comparing the models across all participants, as well as in each group separately. Non-Bold scores reflect the difference with regard to the winning ‘full’ hybrid model with ${\varvec{\uplambda}}$.

*NW* normal-weight, *OW* overweight, *OB* obese, *MB* model-based, *MF* model-free.

### Correcting for age and IQ

To check the robustness of our findings and rule out that the observed group differences could be explained by age^21,48,49^ or IQ^16,21,50,51^ rather than weight status, we reran all models post hoc including age and non-verbal IQ as covariates of no interest. In case of nonparametric tests, the analyses were performed after having regressed out age and non-verbal IQ from the dependent variables using linear regression.

Adding the covariates did not change the results qualitatively—the outcomes were largely in line with the original analyses and suggest that weight status, over and above age and IQ, explains unique variance in the degree to which individuals rely on measures of model-based, and possibly model-free, control (see Supplemental Table S1 for a graphical overview of the outcomes of all analyses of interest). Notably, the reported group differences in model-based control, as observed in stay probabilities, and the relative reliance on model-based and model-free control, as reflected in the model parameter *ω,* were relatively robust when correcting for age and non-verbal IQ. However, the pairwise comparison in model-based control between normal-weight and obese participants did not reach significance. Furthermore, on the continuous level we observed a similar negative relationship between BMI and model-based control (stay probabilities) (see “Supplemental Materials” for statistics).

## Discussion

The aim of this study was to investigate the relationship between weight status (i.e., normal-weight, overweight, and obese) and reliance on model-based and model-free control in the two-step task^16,25,30^. Our results indicate that obese participants relied less strongly on model-based control than overweight and—to a lesser extent—normal-weight participants, with no difference in performance between overweight and normal-weight participants. This was observed in group analysis of participants’ choice behaviour (i.e., stay probabilities), as well as in the continuous analysis where BMI negatively related to model-based choice behaviour. No quadratic relationship with BMI was observed. Furthermore, computational modeling of participants’ choices revealed a similar group difference in the weighting of model-based and model-free control (i.e., *ω*) that was driven by less model-based control for obese relative to overweight and normal-weight participants. Secondary analyses, however, did not show group differences in the slowing of second-stage reaction times after rare transitions, as would be expected given the observed decrease in model-based choice behaviour in obesity.

Although seemingly contradictory, together these findings may in fact suggest that the observed obesity-related difference in model-based control is driven, in part, by enhanced reliance on model-free computations. This interpretation concurs with our post hoc simple effects analyses of stay probabilities, which revealed that the group difference in model-based control was driven by an increased inclination of obese (relative to normal-weight) to stay with their choice specifically after trials on which a rare transition led to reward. Rare trials are the trials of interest in this task, because performance following rare trials is used to dissociate model-based from model-free choices. Common trials, on the other hand, lead to the same decision in model-based and model-free agents. The group difference was only observed for rewarded, not unrewarded rare trials. We speculate that obese individuals may more easily fall back on model-free control, or in other words be more reactive after having been rewarded than normal-weight participants, whilst relying similarly on model-based control in the case of no reward. This speculative interpretation should be interpreted with care, as it has been shown that model-free control on this task can potentially be due to a misunderstanding of the model of the task^54^. Furthermore, the current task is not designed to address this subtle effect, which could explain why it was not reflected in a group difference in model-free control in the analysis of stay probabilities*.*

Our findings are in contrast to those of a previous study by Voon et al.^19^ using the same paradigm. When comparing non-obese controls and obese participants with and without binge-eating disorder, Voon et al.^19^ reported no difference in the weighting parameter *ω* between obese participants *without* binge-eating disorder and non-obese controls, whereas *ω* was on average lower for obese participants *with* binge-eating disorder relative to matched non-obese controls. Interestingly, our findings in healthy obese participants better match the previous findings in obese participants *with* binge-eating disorder. It should be noted however that *ω*, and thus the reliance on model-based over model-free control, was much higher in the current study (mean (SD) omega: 0.6 (0.11) vs. 0.3 (0.24), range 0–1). The discrepancy between the studies can be explained by several factors. First, the current study tested a more severely obese group than the Voon-study with a mean BMI of 35.4 kg/m^2^ (SD 4.5) vs. 31.5 kg/m^2^ (SD 3.6). In fact, in terms of BMI our sample was closer to the binge-eating group (mean BMI[kg/m^2^] 35.0, SD 5.6). It may thus be the case that the reported finding of a lower weighting parameter *ω* in binge-eating disorder in the Voon-study can partially be explained by the severity of obesity. Alternatively, even though no psychiatric conditions were reported, the obese participants in our sample might unbeknownst fulfill criteria for binge-eating disorder or have other co-morbidities, as we did not conduct a full psychiatric screening. We did, however, observe a group difference in self-reported depressive symptom score as assessed by Beck’s Depression Inventory (BDI), with higher—but subclinical—scores for the obese relative to the normal-weight and overweight group. Post hoc analyses showed that variation in BDI scores could not explain the observed group difference in reliance on model-based control. On the continuous level, BDI score did seem to moderate the negative relationship between model-based control and BMI, with a stronger negative relationship for higher BDI scores. It should be noted that the results of the post hoc analyses have to be interpreted with caution because BDI score was not systematically sampled and the use of the BDI as a continuous measure of depressive symptoms in obesity is criticized. The BDI includes both non-somatic and somatic items. High scores on the somatic items (e.g., fatigue, sleep disturbance, body image) may either reflect true depressive symptoms or they are instead related to individuals’ obesity. Second, we included an intermediate weight group for increased sensitivity to detect group differences and potential quadratic effects that might otherwise remain uncovered. The group difference in model-based control in the current study was indeed mostly driven by the difference between overweight and obese participants. We therefore recommend that cognitive studies of obesity should include a wide BMI range, preferably also sampling severe to morbid obesity to assess for quadratic relationships, and to carefully disentangle between contributions of weight status and compulsive measures such as binge-eating symptoms.

The observed difference in reliance on model-based control in obesity generally concurs with previous outcome devaluation studies in relation to obesity that found reduced goal-directed control^13,14^. Goal-directed and model-based control are often equated^11^ and have been found to relate, albeit weakly^15–17^. However, the concepts measured in the two types of tasks do not reflect the exact same constructs. Whereas the two-step task is designed to dissociate model-based and model-free control, it is difficult to disentangle reliance on goal-directed and habitual control in outcome devaluation paradigms in humans. In fact, for the current version of the two-step task—with reliance on model-based vs. model-free choice strategy *not* affecting overall outcome—one could paradoxically speculate that those who rely more strongly on model-free control are putatively even more efficient. For model-based control to be a more sensible strategy than model-free control, it should pay off to spend the extra cognitive resources associated with it^55^. That participants indeed follow this strategy was recently confirmed in a similar sequential decision-making task in which the incentive size was manipulated: model-based control indeed increased with larger incentives in a heterogeneous nonpatient population^56^. Furthermore, goal-directed and habitual control may be organized hierarchically rather than in parallel. That is, the goal-directed system may benefit from habits in goal-pursuit and thus rely on the habit system^57^, and the habit system may affect what goals are selected and pursued by the goal-directed system^58^. Empirical evidence for the existence of such hierarchies comes from a new generation of sequential decision-making tasks^59–61^. It will be relevant for future studies to focus on habitual goal-selection in the context of obesity, as has been suggested for addiction and other disorders of compulsivity^58^, and investigate if it relates more closely to maladaptive eating behaviour in daily life.

The current study has several limitations. First, the dataset was collected in two parts with a sampling bias in terms of group and sex (see Supplemental Figure S1). Due to this bias we could not meaningfully account for sex and sample (2012–2014 vs. 2018) as covariates of no interest, because variance explained by sample and weight group or sample and sex cannot be disentangled in our design^62^. However, the task was identical in both sampling periods and administered in very similar lab spaces within the department. More importantly, extensive computerized instructions were implemented to minimize variability in performance due to differences in instructions between experimenters. We are therefore fairly confident that the observed group differences are not confounded by sampling period. Second, as emphasized above, the observed group differences are subtle with modest effect sizes and await replication. We speculate that these differences may be more pronounced when taking into account participants’ diet rather than obesity. Rodent studies suggest that rather than obesity, the intake of high fat and/or sugar diets may better predict alterations in dopamine-transmission^63–68^. We expect these changes to be at the heart of the maladaptive behavioural control in obesity^24^ and there is accumulating evidence that different measures and manipulations of dopamine transmission overall related positively to model-based control as measured in the two-step task^25–29^. Whether diet rather than obesity relates to maladaptive behavioural control needs to be addressed in further studies. A third limitation is that, although the continuous analyses converge with the observed group differences in model-based control and strengthens the conclusion that obesity is indeed associated with altered reliance on model-based vs. model-free control, the design of the current study was not optimal for this type of analysis. BMI was not equidistributed across the complete sample due to the group-based recruitment-strategy. Hence, the current study might have been underpowered to robustly show true effects between BMI and behavioural control strategies on a continuous level. Another reason for interpreting the reported relationship between BMI and behavioural control on a continuous level with care is the low retest reliability of the task, as has recently been shown in a large-scale investigation of self-regulation paradigms^69^. For the investigation of individual differences in task performance within groups, other variables such as reaction time and latent variables from drift diffusion modeling could give a more reliable estimate of behavioural control^70^. Following Hedge et al.^71^, the poor retest reliability that is the result of low between-subject variability does not negate the observed group differences. In fact, low-between subject variability is required to observe reliable group differences in task performance. Despite these limitations, the findings from our two independent analysis approaches did converge. That is, analysis of raw choice behaviour in terms of stay probabilities and of the model parameter *ω* both point to alterations in the reliance on model-based vs. model-free control in obesity. Simulation recovery analysis of the parameter estimates of the computational models further strengthened our confidence in the observed findings, because it recovered the observed three-way interaction between group, reward and transition probability on stay probabilities.

In conclusion, we found evidence for a relationship between the degree of obesity and reliance on model-based and model-free control relative to overweight and normal-weight participants, which may in fact be linear rather than quadratic in nature. Obesity, on the group-level, was associated with relatively lower model-based control compared to normal-weight and overweight, which was driven by an increased inclination of obese (relative to normal-weight) to stay with their choice specifically after trials on which a rare transition led to reward. Together, our findings suggest that it may be the combination of decreased model-based and increased model-free control in this task that characterizes the obese group. Whether or not the observed effects are dopamine-mediated, as hypothesized, remains an open question that warrants further investigation, for example, by pharmacologically manipulating dopamine transmission, or investigating the interaction between BMI and individual differences in dopamine transmission in terms of genetic or epigenetic variation.

## Supplementary Information

Supplementary Information.

## Data Availability

The datasets analysed during the current study are available from the corresponding author on reasonable request.
