# Author Correction: Reliance on model-based and model-free control in obesity

**DOI:** 10.1038/s41598-021-83028-z

**Published:** 2021-02-11

**Authors:** Lieneke K. Janssen, Florian P. Mahner, Florian Schlagenhauf, Lorenz Deserno, Annette Horstmann

**Affiliations:** 1 Integrated Research and Treatment Center Adiposity Diseases, Leipzig University Medical Center, Leipzig, Germany; 2 Department of Neurology, Max Planck Institute for Human Cognitive and Brain Sciences, Leipzig, Germany; 3 Department of Psychiatry and Psychotherapy, Charité-Universitätsmedizin Berlin, Campus Charité Mitte, Berlin, Germany; 4 Max Planck UCL Centre for Computational Psychiatry and Ageing Research, University College London, London, UK; 5 The Wellcome Centre for Human Neuroimaging, University College London, London, UK; 6 Department of Child and Adolescent Psychiatry, Psychotherapy and Psychosomatics, University of Würzburg, Würzburg, Germany; 7 Department of Psychology and Logopedics, Faculty of Medicine, University of Helsinki, Helsinki, Finland

Correction to: *Scientific Reports* 10.1038/s41598-020-79929-0, published online 31 December 2020

The version of this Article previously published contained lower resolution images for Figures 1, 2 and 3.

In addition, in Figure 2 and 3 the data points in the box plots have changed their position (within each group) due to a rerun of the code used to generate the plots. These changes do not affect the overall conclusions of the Article.

These errors have now been corrected in the PDF and HTML versions of the Article.

