# Supplementary material for: Reliance on model-based and model-free control in obesity
Source: Sci Rep. 2020 Dec 31;10:22433. doi: 10.1038/s41598-020-79929-0 (PMC7775466; doi:10.1038/s41598-020-79929-0)
Supplement: Supplementary file 1 — Supplementary Information. [file 41598_2020_79929_MOESM1_ESM.docx]

**Supplemental Materials**

**Reliance on model-based and model-free control in obesity**

Lieneke K. Janssen, Florian P. Mahner, Florian Schlagenhauf,
Lorenz Deserno, Annette Horstmann

**Supplemental Table 1. Overview of the outcomes of the analyses of interest. The study was designed primarily for group-based analysis (left column), but also permitted secondary continuous analysis of BMI (right column). For each measure, the original analysis is reported as well as the covariate analysis with covariates age and non-verbal IQ.**

|  |  | **Group-based (primary)** | | **Continuous (secondary)** | |
| --- | --- | --- | --- | --- | --- |
| **Behavioral** | | | |  |  |
| Stay probabilities | | | |  |  |
| Interaction  Reward x Transition | MB | ANOVA | OB<[OW,NW] | BMI & BMI^2^ | negative linear |
|  |  | with covariates | ^^^  OB<[OW,NW^^^] | with covariates | negative linear |
| Main effect Reward | MF | ANOVA | X | BMI & BMI^2^ | X |
|  |  | with covariates | X | with covariates | X |
| **Computational modelling** | | | |  |  |
| ω | MB vs. MF | ANOVA | OB<[OW,NW] | BMI & BMI^2^ | negative linear |
|  |  | with covariates | OB<OW | with covariates | X |

*MB = model-based*

*MF = model-free*

*NW = normal-weight*

*OW = overweight*

*OB = obese*

*= a statistical difference is observed*

*X = no statistical difference is observed*

*^^^ observed difference at trend level*

***Post hoc* covariate analysis**

**Correcting for group differences in BDI**

To rule out that the observed effects of interest could be explained by the group difference in self-reported number of depressive symptoms on Beck’s Depression Inventory (BDI), we reran all group-based (primary) and continuous (secondary) analyses *post hoc* including BDI score as a covariate.

The primary effects of interest were robust when correcting for BDI score. That is, the group difference in model-based control as observed in stay probabilities remained significant (Group x Reward x Transition interaction: *F*(2,86) = 3.4, *p* = .037, *η_p_^2^* = .074). This effect was again driven by a larger Reward x Transition interaction term for overweight relative to obese participants (*p* = .014), as well as for normal-weight relative to obese participants (*p* = .040), but not for normal-weight relative to overweight participants (*p* = .891). Also, the absence of a group difference in model-free control in terms of stay probabilities was unaltered (Group x Reward interaction: *F*(2,86) = 2.2, *p* = .116, *η_p_^2^* = .049). Finally, the group difference in relative reliance on model-based and model-free control – as reflected in the model parameter *ω* - was still significant (*F*(2,86) = 4.5, *p* = .013, *η_p_^2^* = .095) and driven by lower reliance on model-based vs. model-free control for obese relative to overweight individuals (*p* = .013). In contrast to the original analysis, the difference between obese and normal-weight participants was only a trend (*p* = .093).

The secondary effects of interest did change significantly when controlling for BDI score. In the original analyses, we observed linear, not quadratic relationships between BMI and the model-based measures of interest (i.e., for the interaction term of Reward x State on stay probabilities, and for model parameter *ω*). Therefore, we only included the linear BMI term in these models and added BDI score as well as the interaction between BMI and BDI score. The interaction term was added because of the reported group difference in BDI score in the main text (**Table 1**). The observed negative linear relationship between BMI and the Reward x State interaction was no longer significant and may be explained away by the interaction between BMI and BDI score at trend level (*β_BDI_* = .918, *p* = .097; *β_BMI_* = .054, *p* = .788; *β_BDI*BMI_* = -1.13 , *p* = .069; adjusted *R^2^* = .088, *F*(3,86) = 3.9, *p* = .012). Similarly, the observed negative linear relationship between BMI and *ω* is no longer observed. Instead we observe a significant interaction between BMI and BDI score (*β_BDI_* = 1.05, *p* = .061; *β_BMI_* = .149, *p* = .465; *β_BDI*BMI_* = -1.28 , *p* = .041; adjusted *R^2^* = .072, *F*(3,86) = 3.3, *p* = .024). In both models, the BMI and BDI interaction is driven by a numerically stronger negative relationship between BMI and the interaction term for higher BDI scores.

**Correcting for age and IQ**

See **Supplemental Table 1** for a graphical overview of the outcomes of all analyses of interest. We found that the reported group differences in model-based control as observed in stay probabilities and the relative reliance on model-based and model-free control as reflected in the model parameter *ω* were robust when correcting for age and non-verbal IQ. That is, we still observed a Group x Reward x Transition interaction at trend level (*F* (2,85) = 2.6, *p* = .080, *η_p_^2^* = .058), that was driven by a larger interaction term for overweight relative to obese participants (*p* = .048), and a similar trend for normal-weight relative to obese participants (*p* = .052). The three-way interaction was again complemented by the continuous analysis, which showed a negative linear, but no quadratic relationship between BMI and the Reward x Transition interaction term (*β_BMI_* = -.533, *p* = .018; *β_BMI_^2^* = .143, *p* = .512, adjusted *R^2^* = .141, *F*(4,85) = 4.7, *p* = .002). Also the absence of a group difference on model-free control in terms of stay probabilities was unaltered, as no Group x Reward interaction was observed (*F* (2,85) = 2.3, *p* = .121, *η_p_^2^* = .048), nor a significant relationship between BMI and the main effect of reward in continuous analysis (*β_BMI_* = .083, *p* = .456; *β_BMI_^2^* = .006, *p* = .956, adjusted *R^2^* = -.030, *F* (4,85) = 0.3, *p* = .844). Furthermore, the group difference in *ω* was still significant (*F*(2,85) = 3.3, *p* = .044, *η_p_^2^ = .*071) and was driven by lower reliance on model-based vs. model-free control for obese relative to overweight individuals (*p* = .013). In contrast to the original analysis, no significant difference was observed between obese and normal-weight participants (*p* = .119). On the continuous level, the linear relationship between BMI and *ω* was no longer significant when adding the covariates (*β_BMI_* = -.17, *p* = .102; *β_BMI_^2^* = -.053, *p* = .605, adjusted R^2^ = .111, *F* (4,85) = 3.8, p = .007).

**Supplemental Figure 1. Overview of participants per group for the two test time frames. A large part of the dataset was acquired between 2012 and 2014** ^1,2^ **and consisted predominantly of normal-weight and overweight participants. Data acquisition was finally completed in 2018 by testing the remaining obese and overweight participants.**

**
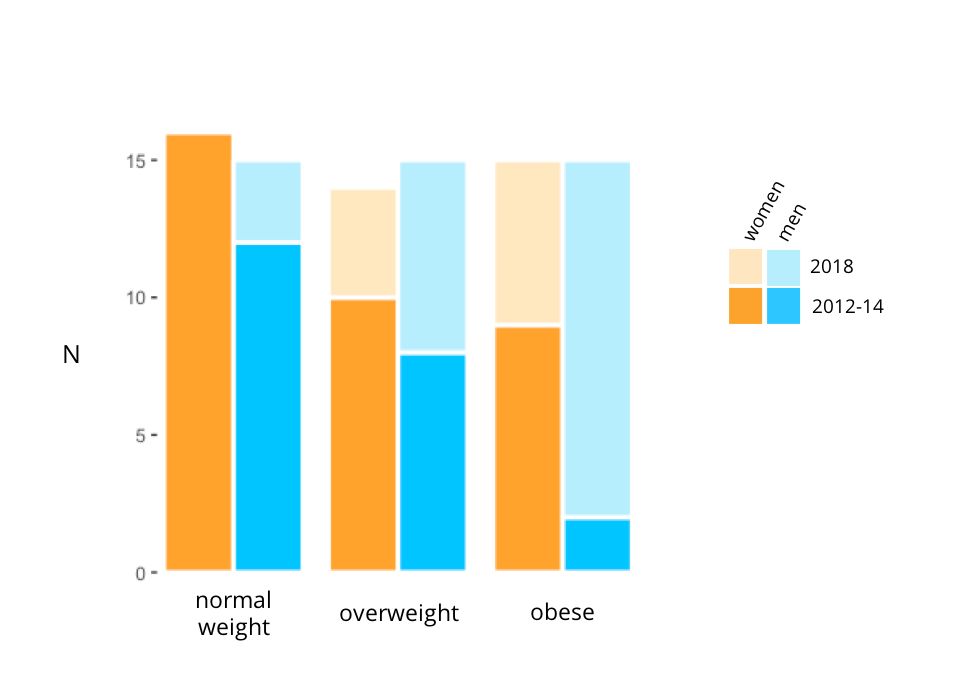
**

**Supplemental Figure 2. Observed average stay probabilities (a) and the stay probabilities simulated based on the parameters of the model (b).**

**a. Observed data**


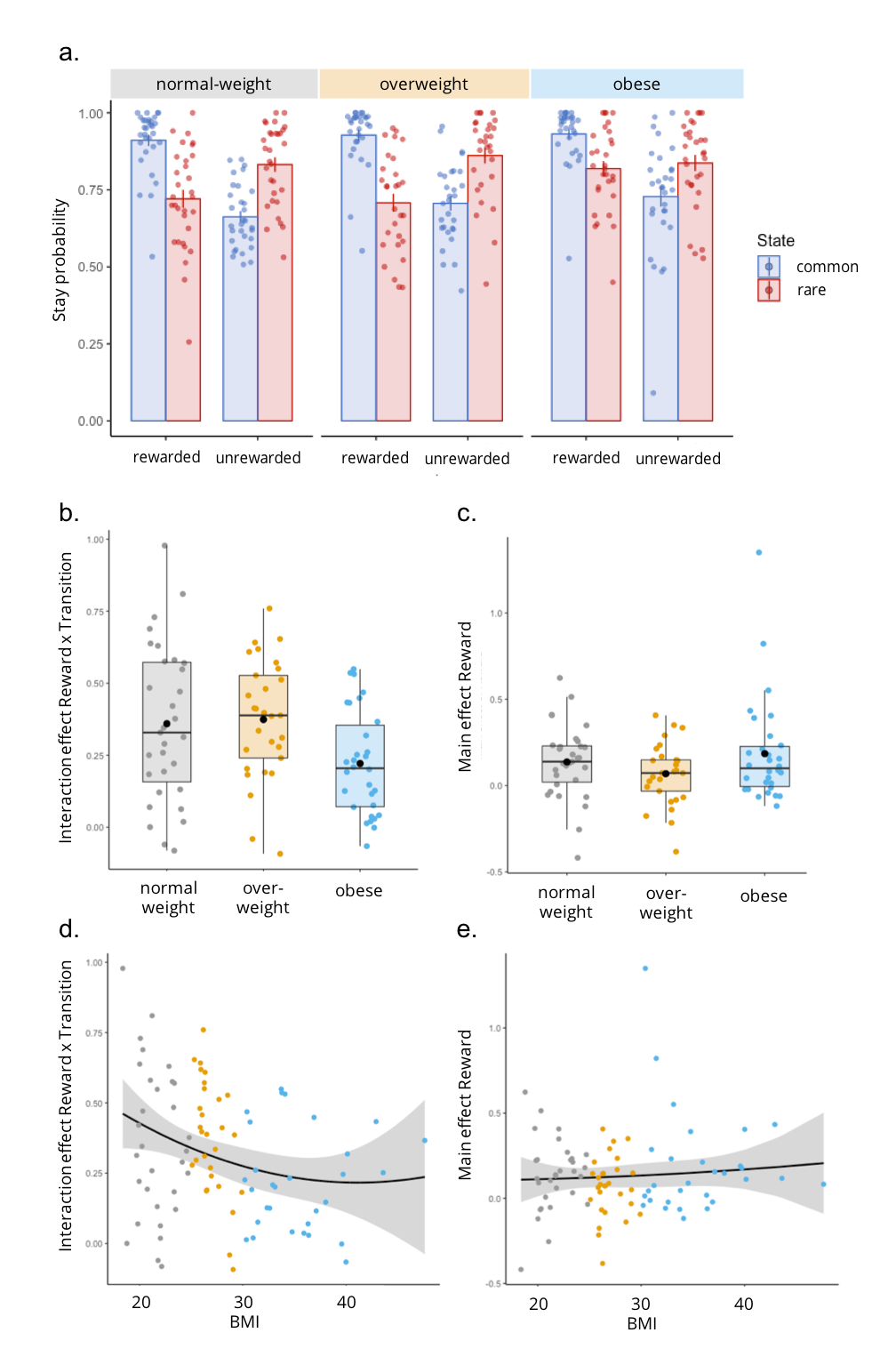


**b. Simulated data**

**
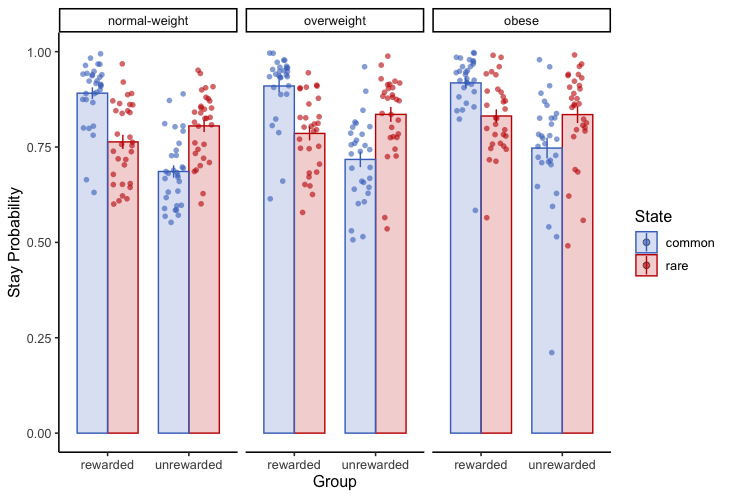
**

**Supplemental references**

1. Sjoerds, Z. *et al.* Slips of Action and Sequential Decisions: A Cross-Validation Study of Tasks Assessing Habitual and Goal-Directed Action Control . *Frontiers in Behavioral Neuroscience*  **10**, 234 (2016).

2. Dietrich, A., de Wit, S. & Horstmann, A. General Habit Propensity Relates to the Sensation Seeking Subdomain of Impulsivity But Not Obesity . *Frontiers in Behavioral Neuroscience*  **10**, 213 (2016).
